# Preoperative Gut Microbiome in Patients With Colorectal Cancer: Potential for Fecal Biomarker–Based Recurrence Risk Prediction

**DOI:** 10.1200/OA-25-00042

**Published:** 2026-01-13

**Authors:** Anne E. Petersen, Konstantina Zafeiropoulou, Mohammed Ghiboub, Claire P.M. van Helsdingen, Joop L.M. Konsten, Nicole D. Bouvy, Jan H.M.B. Stoot, Pieter J. Tanis, Wouter J. de Jonge, Joep P.M. Derikx

**Affiliations:** 1 Department of Pediatric Surgery, Emma Children’s Hospital, Amsterdam University Medical Centers, University of Amsterdam, Amsterdam, the Netherlands; 2 Amsterdam Gastroenterology Endocrinology Metabolism Institute, Amsterdam University Medical Centers, Amsterdam, the Netherlands; 3 Tytgat Institute for Liver and Intestinal Research, Amsterdam UMC, University of Amsterdam, Amsterdam, the Netherlands; 4 Department of Surgery, Viecuri Hospital, Venlo, the Netherlands; 5 Department of Surgery, Maastricht University Medical Center, Maastricht, the Netherlands; 6 Department of Surgery, Zuyderland Medical Center, Heerlen and Sittard-Geleen, the Netherlands; 7 Department of Oncological and Gastrointestinal Surgery, Erasmus Medical Center, Rotterdam, the Netherlands; 8 Department of Surgery, University of Bonn, Bonn, Germany

## Abstract

**Purpose:**

While gut microbiome dysbiosis is known to play a role in colorectal cancer (CRC) initiation and progression, its role in CRC recurrence remains unclear. This study investigates whether the gut microbiome is associated with CRC recurrence.

**Patients and Methods:**

In a prospective observational cohort, preoperative fecal samples from patients with stage I to III CRC undergoing surgical resection were analyzed using 16S rRNA gene sequencing. Alpha diversity and beta diversity were compared between patients with and without recurrence, and differential abundance analyses were conducted to identify bacterial genera associated with recurrence risk.

**Results:**

Among 294 patients, 61 (21%) patients developed recurrence during a median follow-up of 56 months, with a median time to recurrence of 19 months. Alpha diversity did not differ between groups, but beta diversity analysis revealed significantly distinct microbial clustering in patients with recurrence, particularly those with locoregional recurrence. Differential abundance analysis identified five bacterial genera associated with locoregional recurrence (*Acidaminococcus, Alloprevotella, Butyrivibrio, Ruminococcaceae CAG-352*, and *Lachnospiraceae UCG-003*), one with distant recurrence (*Megamonas*), and two with overall recurrence (*Anaeroplasma, Porphyromonas*). Stratifying patients into high- and low-abundance subgroups revealed that those with a high relative abundance of *Porphyromonas* had an increased risk of overall recurrence (hazard ratio, 2.80 [95% CI, 1.54 to 5.10]).

**Conclusion:**

Patients with CRC who develop locoregional recurrence exhibit a distinct preoperative fecal microbial composition compared with those without recurrence. Our findings provide novel insights into the role of the intestinal microenvironment in recurrence and identify *Porphyromonas* as a potential fecal biomarker for overall recurrence risk.

In patients with nonmetastatic colorectal cancer (CRC), disease recurrence is the leading cause of mortality.^1^ Recurrence after curative-intent surgery can occur at the primary tumor site, in regional lymph nodes, and at distant sites. Several risk factors for CRC recurrence have been identified, primarily focusing on tumor histopathology and clinical features.^2^ In clinical practice, the tumor node metastasis (TNM) staging system, accompanied by adverse tumor features such as extramural venous invasion (EMVI), serves as a primary framework for prognosis prediction.^3,4^ Nevertheless, there is a need for improvement of the current accuracy of existing prediction tools for CRC recurrence.^5^

Limited predictive accuracy likely reflects an incomplete understanding of the underlying mechanisms driving recurrence, including potential contributions from interactions with environmental factors such as the microbiome. Gut microbiome dysbiosis has emerged as a key component in CRC initiation and progression.^6^ However, its involvement in the development of recurrence after curative-intent surgery remains largely unexplored.^7^ A comprehensive understanding of preoperative gut microbiome profiling may provide valuable mechanistic insights into CRC recurrence with potential for identifying predictive biomarkers for recurrence and therapeutic targets. Therefore, in a prospective observational CRC resection cohort,^8^ we assessed whether the gut microbiome is associated with disease recurrence.

Detailed methods are provided in Appendix 1. Briefly, 385 patients with stage I to III CRC at diagnosis who underwent surgical resection were screened. Ninety-one were excluded because of the lack of a fecal sample (n = 87) or antibiotic use at the time of sampling (n = 4). Stage IV cases were excluded as their recurrence risk is inherently high, and we consider it unlikely to be substantially influenced by microbiome-related factors.

The final cohort included 294 patients. The overall recurrence rate was 21% (61 of 294 patients). At the time of initial diagnosis of recurrence, 11 patients had only locoregional recurrence, 10 patients had combined locoregional and distant recurrence, and 40 patients had only distant recurrence. The median time to initial diagnosis of recurrence was 19.0 (IQR, 11.1-28.1) months. The median follow-up duration for all patients was 56 (IQR, 47.2-61.2) months. Clinical and demographic characteristics were comparable between patients who developed recurrence and those who did not (Table 1). However, among pathologic characteristics, the higher TNM stage, poorer tumor differentiation, and the presence of EMVI were positively associated with recurrence.

To assess potential selection bias because of missing preoperative fecal sample or antibiotic use during sampling, we compared baseline clinical and pathologic characteristics between included (N = 294) and excluded patients (n = 91). No differences were found in baseline characteristics.

Next, using 16S rRNA gene sequencing, we analyzed the microbial composition of fecal samples collected 1 day before the primary surgery. Comparisons were made between patients with and without recurrence and further stratified by recurrence location (locoregional (including distant) or distant alone). A detailed description of the statistical analysis pipeline is provided in Appendix 1.

Alpha diversity analysis revealed no differences in microbial richness and Shannon diversity index between patients with (n = 61) and without recurrence (n = 233, Fig 1A). However, principal coordinate analysis showed that patients who developed recurrence clustered differently from those who did not, explaining 0.5% of the variance in microbial composition (R^2^ = 0.005, Fig 1B). Subgroup analyses indicated that this clustering was primarily observed in patients with locoregional recurrence (n = 21), who exhibited distinct microbial profiles compared with patients without recurrence (R^2^ = 0.006, Fig 1D). Patients with only distant recurrence (n = 40) did not show significant clustering (R^2^ = 0.004, Appendix Fig A1), suggesting that the difference in microbial composition between patients with and without recurrence is mainly determined by the subgroup of locoregional recurrences.

Differential analysis of genus-level relative abundances using DESeq2^9^ showed that patients with recurrence had lower levels of several genera compared with patients without recurrence (Fig 1E). To assess whether these differences were influenced by low abundance or outlier samples, we plotted their relative abundance distributions as violin plots, combining box plot summaries with kernel density estimation (Appendix Fig A2).

*Anaeroplasma* was reduced in both locoregional (log_2_ fold change = –2.12) and distant recurrence (log_2_ fold change = –1.39), whereas *Porphyromonas* was elevated (log_2_ fold change = 2.52 and 0.83, respectively). These two genera were the only ones statistically significantly associated with both recurrence types and were therefore selected for patient stratification. Other genera in Figure 1E were significant in only one comparison and were excluded from subsequent biomarker identification steps, that is, Kaplan-Meier analysis. We assessed *Porphyromonas* and *Anaeroplasma* individually rather than as a ratio, to avoid combining opposing trends, and summarized their distributions in violin plots (Appendix Fig A2) and descriptive statistics. Overall, the median relative abundance was 0% for both genera. Mean relative abundances (±SD) for *Porphyromonas* were 0.13 ± 0.74% across all patients, 0.09 ± 0.41% in distant recurrence, 0.73 ± 1.62% in locoregional recurrence, and 0.08 ± 0.64% in nonrecurrence. For *Anaeroplasma*, mean abundances were 0.10 ± 0.50% overall, 0.05 ± 0.23% in distant recurrence, 0.18 ± 0.79% in locoregional recurrence, and 0.10 ± 0.51% in nonrecurrence.

Based on the relative abundances of *Porphyromonas* and *Anaeroplasma*, patients were stratified into high- and low-abundance subgroups. High-abundance groups were defined as the top 10% of values (>0.03% for *Porphyromonas*, >0.10% for *Anaeroplasma*; see Thresholds definition in Appendix 1). Patients with a high preoperative relative abundance of *Porphyromonas* had an increased risk of recurrence (hazard ratio [HR], 2.80 [95% CI, 1.54 to 5.10]; *P* < .005, Fig 1F), whereas low *Anaeroplasma* abundance was not associated with recurrence risk (*P* = .267, Appendix Fig A1E). In stratified analyses, *Porphyromonas* abundance did not differ across tumor differentiation grades, but was significantly higher in EMVI-positive cases (Appendix Fig A3), suggesting that its association with recurrence may involve EMVI.

A key finding of our study is that overall differences in the microbial community structure (beta diversity) were observed only in patients who later developed locoregional recurrence. Although a clear definition for locoregional recurrence of CRC is lacking, this novel observation supports the hypothesis that the gut microbiome may influence the survival and implantation of perianastomotic tumor cells. Several individual taxa were also associated with overall recurrence, which also includes distant recurrences, and could reflect systemic effects of specific bacteria or their association with adverse tumor characteristics that inherently increase recurrence risk. In particular, a high preoperative fecal abundance of *Porphyromonas* increased the risk of overall recurrence. This periodontal pathogen has been implicated in proinflammatory processes^10^ and correlates with various systemic diseases^11^ and initiation and progression of colorectal and esophageal cancers.^12^ *Porphyromonas* may drive CRC recurrence through induction of cellular senescence^6^ or through negatively affecting tumor cytotoxicity in invariant natural killer T cells.^13^ These hypotheses are based on previously reported roles of these bacteria in inflammation and immune modulation, but confirming them will require further functional and host immune studies. Future studies incorporating functional microbiome assays and host immune profiling will be essential for substantiating these potential links.

Interpretation at the genus level should be approached with caution as intragenus heterogeneity may mask opposing biological functions. Similarly, we emphasize that 16S rRNA gene sequencing data are hypothesis-generating rather than definitive. For instance, the genus *Porphyromonas* includes both commensal and pathogenic species with distinct functional roles.^14^ To investigate species-level associations, we conducted exploratory shotgun metagenomic sequencing on a representative subset of our cohort (21% of patients without recurrence, 17% with distant recurrence, and 15% with locoregional recurrence). This analysis confirmed the presence of multiple *Porphyromonas* species associated with recurrence, including *P. asaccharolytica* and *P. somerae*, and an uncharacterized bin (SGB1983). While this subset provides higher taxonomic resolution, the sample size was insufficient for differential abundance analysis. Expanding metagenomic profiling to the full cohort will be essential for validating these findings, uncover species-specific patterns, and clarify the role of this genus in CRC recurrence.

To identify differentially abundant genera, we used DESeq2, a widely applied method in microbiome research. Acknowledging its limitations with sparse data,^15^ we complemented it with linear discriminant analysis effect size (LEfSe). Both methods identified *Porphyromonas* as enriched in the recurrence groups, with LEfSe confirming statistical significance in local recurrence versus non-recurrence (linear discriminant analysis [LDA], 3.69, adjusted *P* = .044) and distant recurrence versus non-recurrence (LDA, 2.79, adjusted *P* = .027), reinforcing the robustness of this finding. LEfSe also detected the family Acidaminococcaceae as enriched in the nonrecurrence group compared with local recurrence (LDA, 3.77, adjusted *P* = .045), in line with our DESeq2 results at the genus level for *Acidaminococcus*.

This study has a couple of limitations that warrant consideration. The exclusion of 91 patients because of missing fecal samples or antibiotic use might have introduced selection bias. However, since no significant differences were observed in clinical or pathologic features between included and excluded patients, any bias from this exclusion is likely limited. Another limitation is the reliance on a single preoperative time point for sampling. The composition of the gut microbiome is dynamic and subject to temporal and spatial variation, and the extent to which a single preoperative fecal sample reflects the perianastomotic microbiome over time remains uncertain.

Nevertheless, fecal microbiome analysis remains a practical and noninvasive method for assessing gut microbiota. If future studies validate the prognostic relevance of fecal *Porphyromonas* as a predictive biomarker in larger cohorts, this methodology could be easily integrated into clinical practice for recurrence risk stratification and contribute to more patient-tailored follow-up strategies. Moreover, identification of bacterial genera that are associated with a decreased risk of locoregional recurrence may provide a foundation for exploring whether microbiome modulation could play a role in reducing recurrence risk.

## Supplementary Material

Supplementary figure 1

Supplementary figure 2

Supplementary figure 3

Appendix

## Figures and Tables

**Fig 1:**
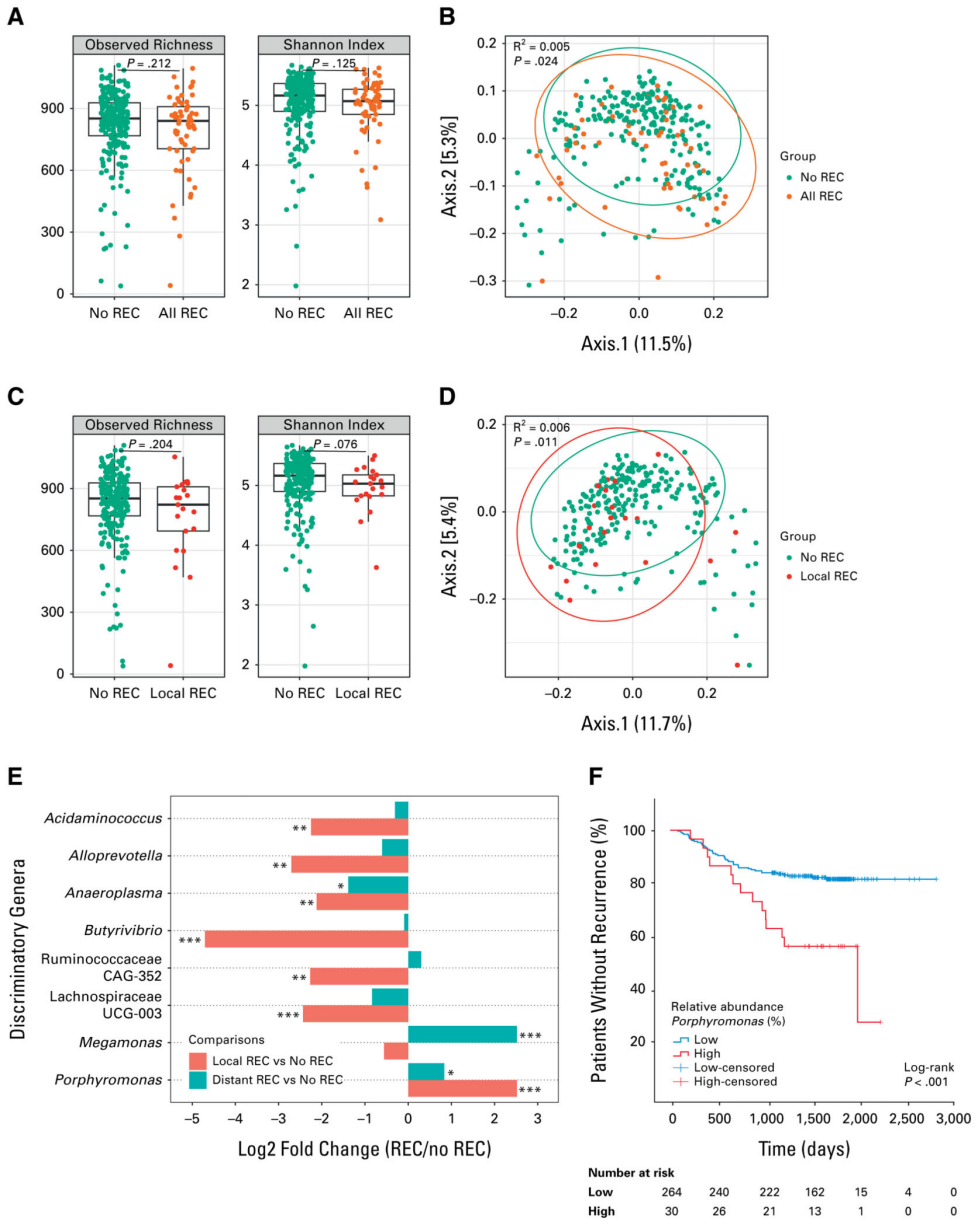
(A) No difference in preoperative fecal alpha diversity between patients with and without recurrence, as depicted by the observed richness and Shannon diversity index. Dots represent fecal samples, colored by recurrence status. (B) Patients with recurrence cluster different from those who did not. (C) No difference in alpha diversity between patients with locoregional recurrence and without recurrence. (D) Patients with locoregional recurrence cluster different from those without recurrence. (E) Differentially abundant genera between patients with and without recurrence. (F) Kaplan-Meier curve of overall recurrence risk based on high or low preoperative fecal abundance of *Porphyromonas*. Patients with a high fecal level of *Porphyromonas* are at increased recurrence risk. Vertical marks indicate censored patients at the end of the follow-up period. All REC, patients with recurrence; local REC, patients with locoregional recurrence; no REC, patients without recurrence; REC, recurrence. *P-adjusted < .05; **P-adjusted < .01; ***P-adjusted < .001.

**Table 1:** Patient Characteristics

Variable	Total (N = 294)	No Recurrence (n = 233)	Recurrence (n = 61)	*P*
Age, years, median (IQR)	67 (59-74)	68 (60-74)	64 (57-73)	.071
Sex, female, No. (%)	119 (40.6)	99 (42.5)	20 (32.8)	.189
Neoadjuvant therapy, No. (%)	31 (10.5)	26 (11.1)	5 (8.2)	.502
Tumor location, No. (%)				.605
Colon	234 (79.6)	184 (79.0)	50 (82.0)	
Rectum	60 (20.4)	49 (21.0)	11 (18.0)	
TNM stage, No. (%)				.008
I	79 (26.9)	73 (31.3)	6 (9.8)	
II	72 (24.5)	52 (22.3)	20 (32.8)	
III	143 (48.6)	108 (46.4)	35 (57.4)	
Tumor size, cm, median (IQR)	3.0 (2.2-4.8)	3.0 (2.2-4.5)	3.5 (2.5-5.0)	.093
Tumor differentiation, No. (%)				.006
Undifferentiated/poorly	27 (9.2)	18(7.7)	9 (14.8)	
Moderately	42 (14.3)	29 (12.4)	1 3 (21.3)	
Moderately well	220 (74.8)	181 (77.6)	39 (63.9)	
Well	5 (1.7)	5(2.1)	0(0)	
EMVI, positive, No. (%)	55 (18.7)	33 (14.2)	22 (36.1)	<.001
Lymphovascular invasion, positive, No. (%)	63 (21.4)	46 (20.5)	17 (28.3)	.197
Perineuralinvasion, positive, No. (%)	13(11.6)	6 (7.5)	7 (21.9)	.048
R1, positive, No. (%)	1 (0.3)	0(0)	1 (1.6)	.130
Mismatch repair deficiency, No. (%)				.022
Yes	29 (9.9)	24 (10.3)	5 (8.2)	
No	212 (72.1)	160 (68.7)	52 (85.2)	
Not assessed	53 (18.0)	49 (21.0)	4 (6.6)	
Microsatellite instability, No. (%)				.170
Yes	23 (7.8)	19 (8.2)	4 (6.6)	
No	20 (6.8)	19 (8.2)	1 (1.6)	
Not assessed	251 (85.4)	195 (83.7)	56 (91.8)	

Abbreviations: EMVI, extramural venous invasion; R1, microscopically tumor-positive resection margin; TNM, tumor node metastasis.

## Data Availability

A data sharing statement provided by the authors is available with this article at DOI https://doi.org/10.1200/OA-25-00042.
