# Supplementary figures and images for: Preoperative Gut Microbiome in Patients With Colorectal Cancer: Potential for Fecal Biomarker–Based Recurrence Risk Prediction
Source: JCO Oncol Adv. Author manuscript; Available in PMC 2026 Apr 10. (PMC7618991; doi:10.1200/OA-25-00042)

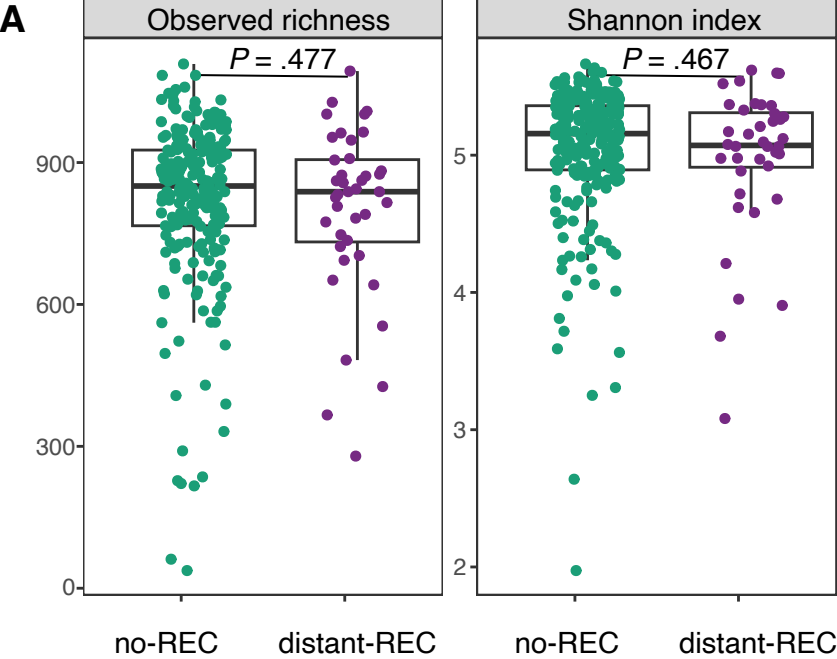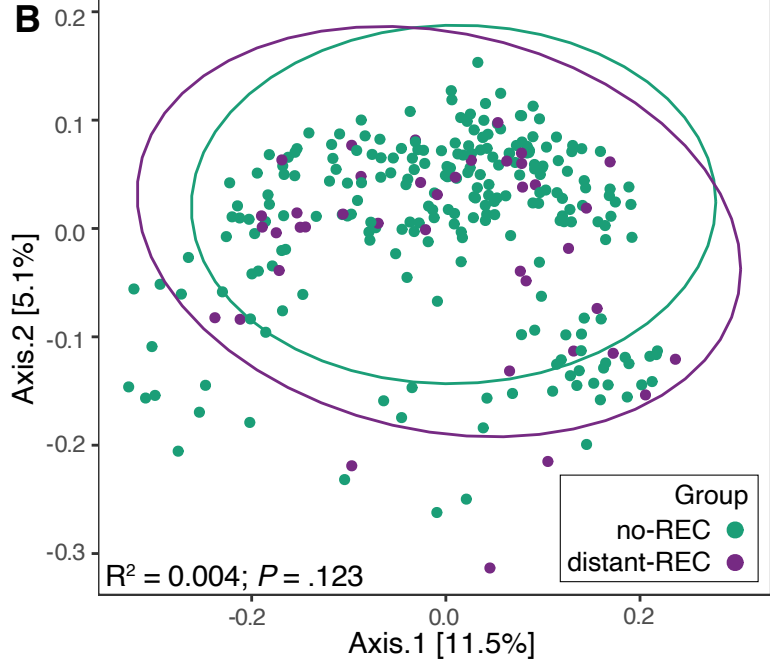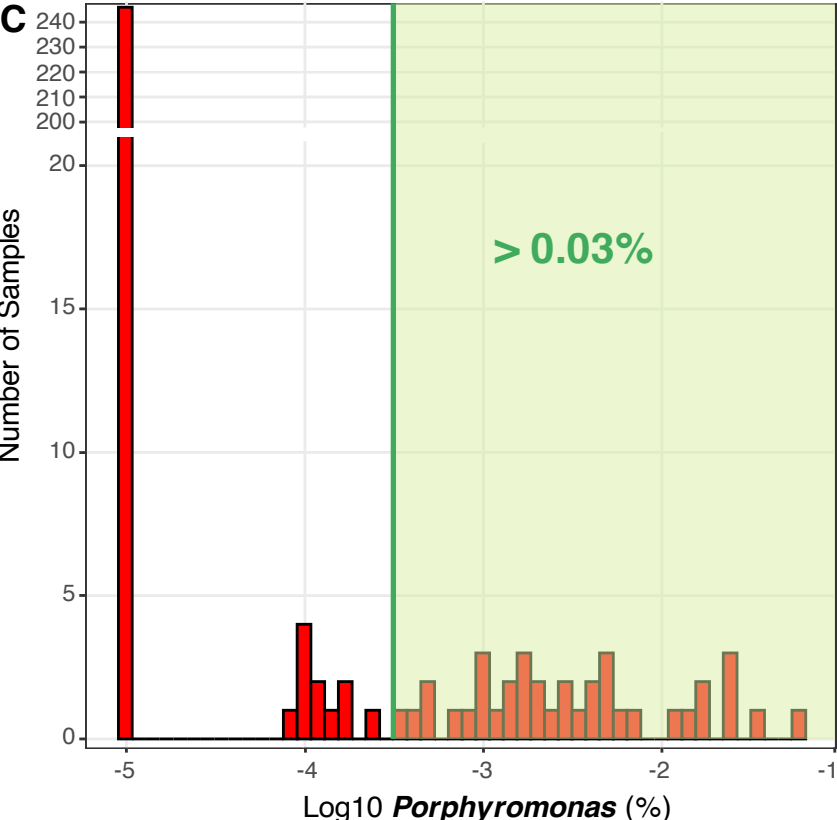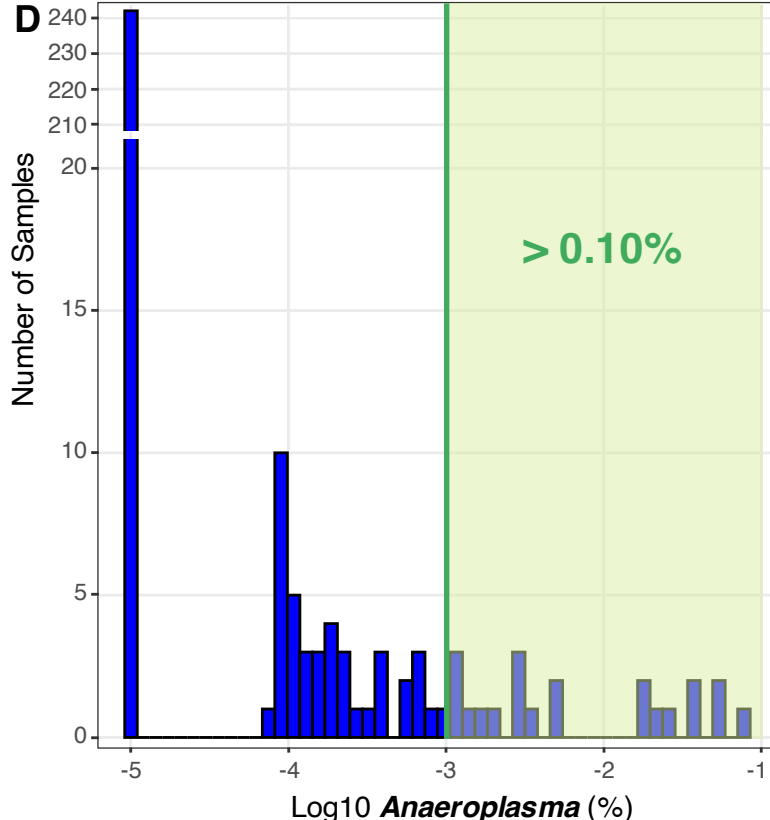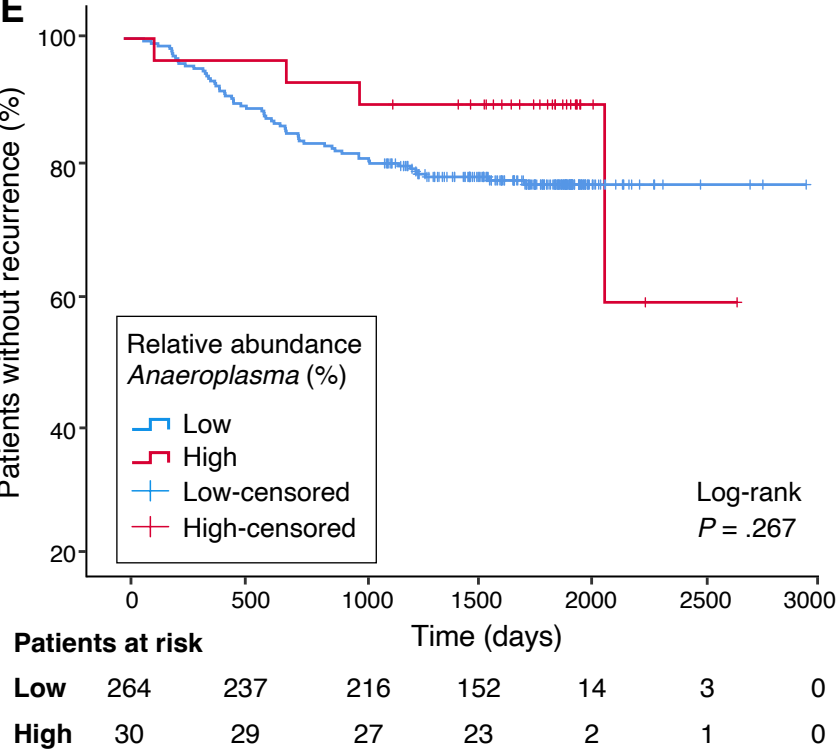

Supplement: Supplementary figure 1 [file EMS212615-supplement-Supplementary_figure_1.pdf]

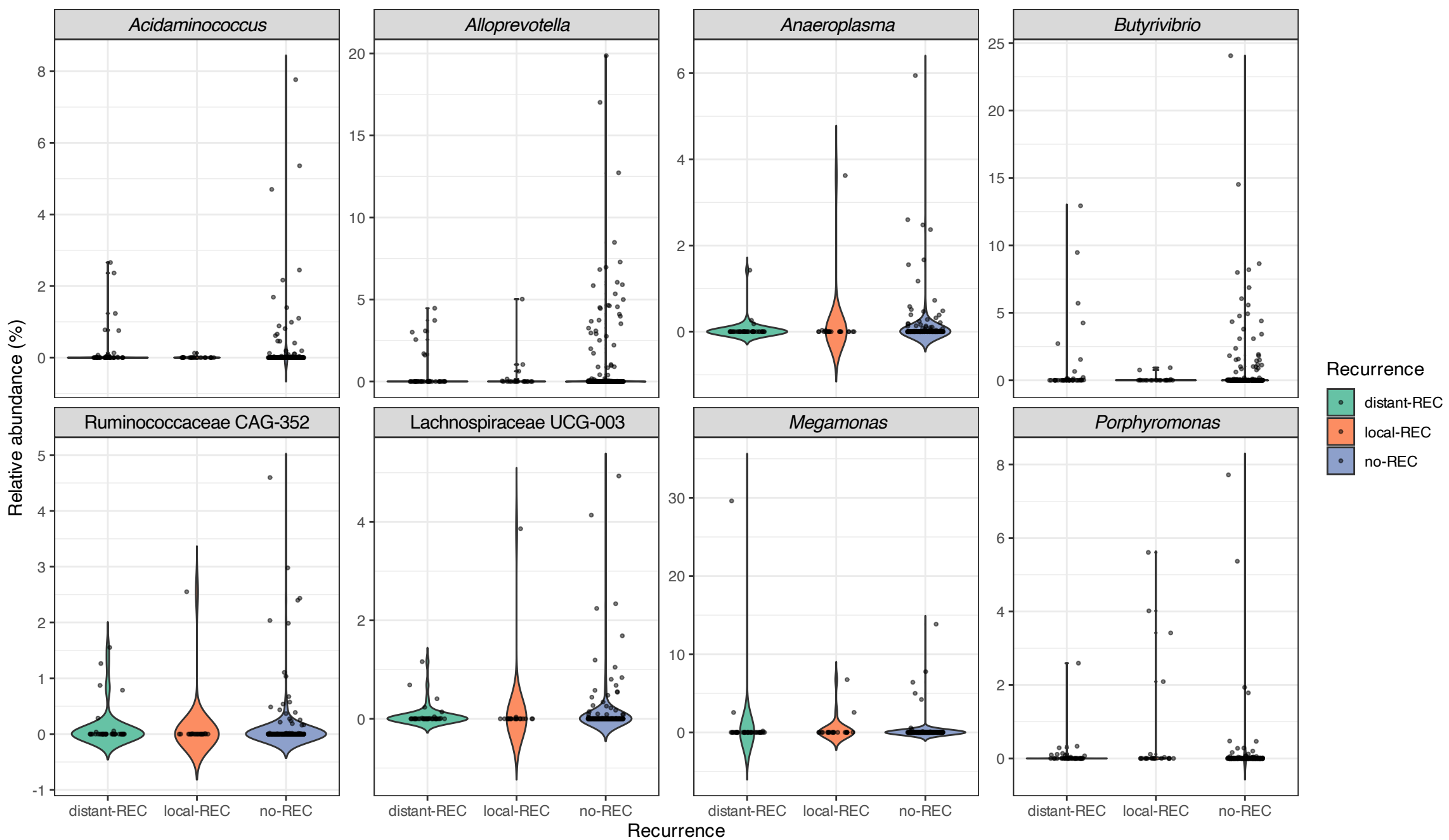

Supplement: Supplementary figure 2 [file EMS212615-supplement-Supplementary_figure_2.pdf]

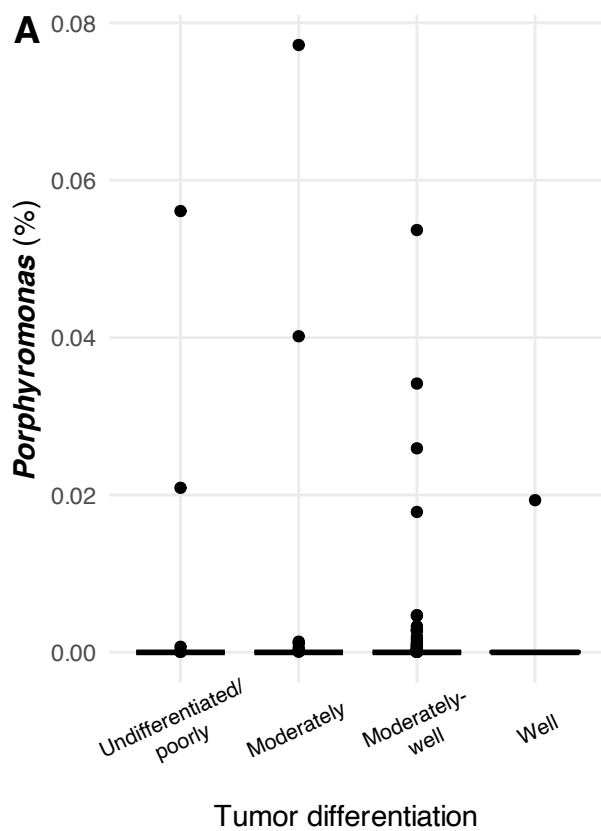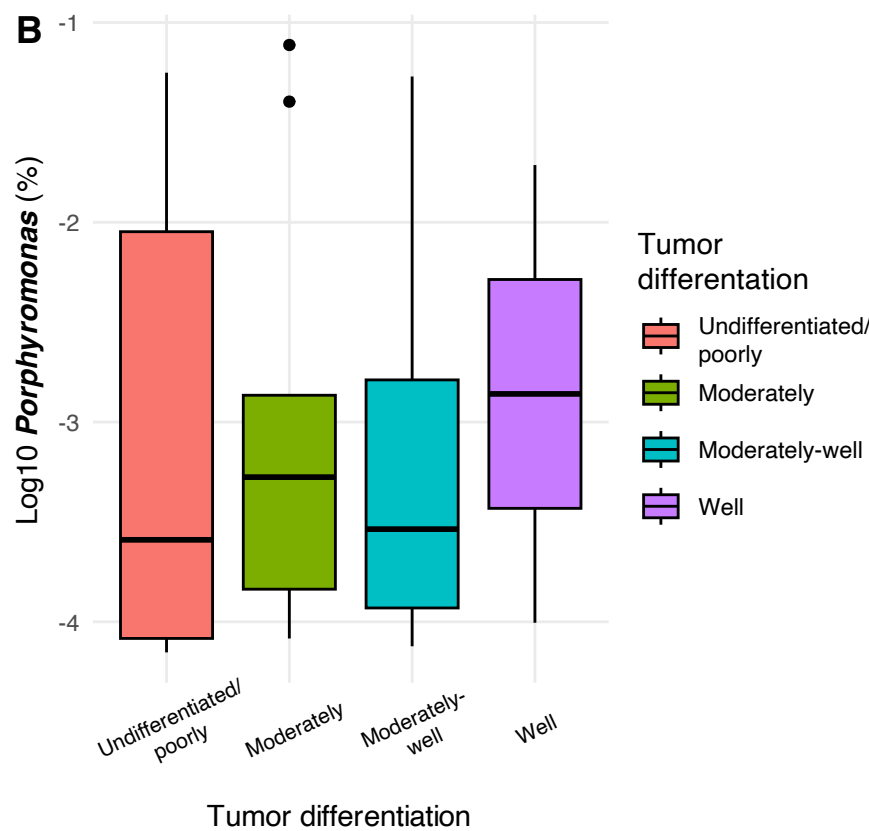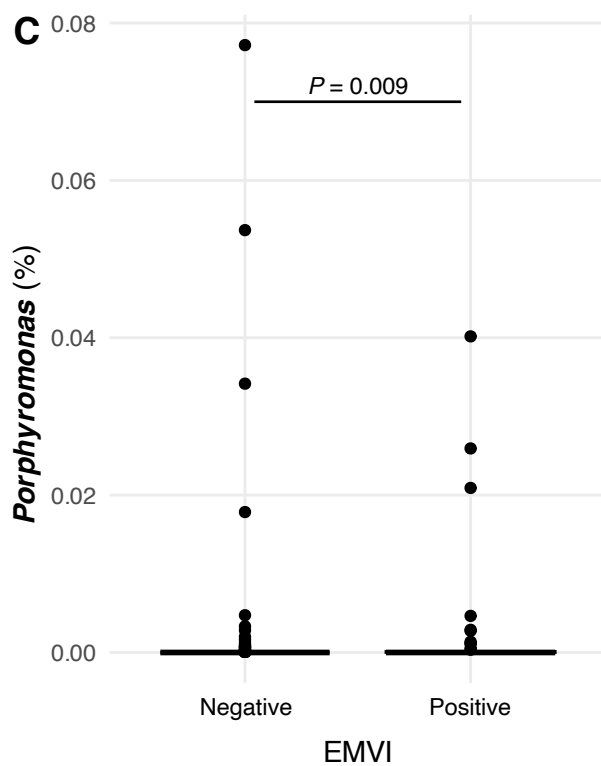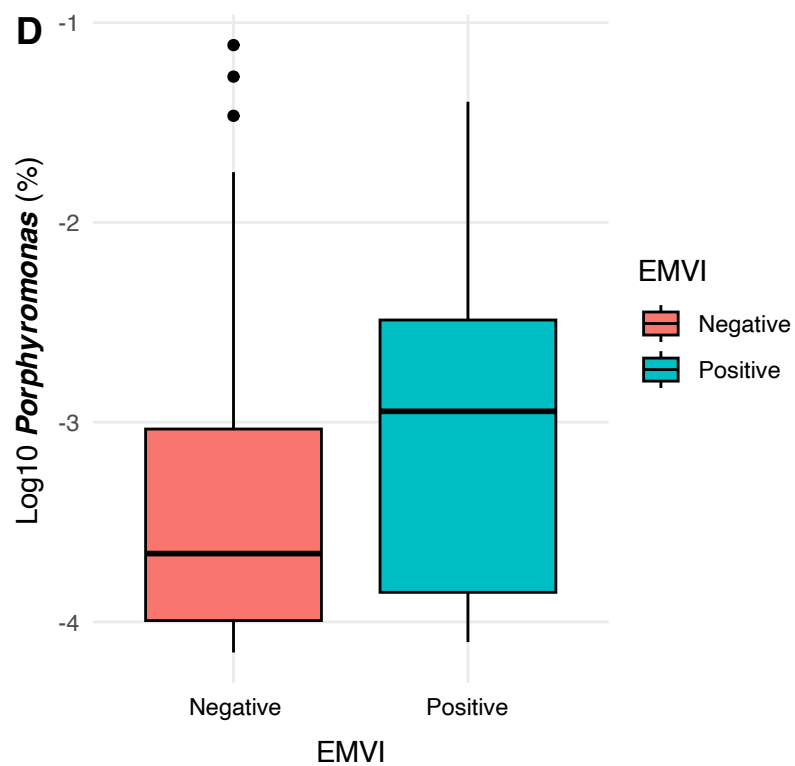

Supplement: Supplementary figure 3 [file EMS212615-supplement-Supplementary_figure_3.pdf]
