# Supplementary material for: Preoperative Gut Microbiome in Patients With Colorectal Cancer: Potential for Fecal Biomarker–Based Recurrence Risk Prediction
Source: JCO Oncol Adv. Author manuscript; Available in PMC 2026 Apr 10. (PMC7618991; doi:10.1200/OA-25-00042)
Supplement: Appendix [file EMS212615-supplement-Appendix.pdf]

Downloaded from ascopubs.org by Universiteit van Amsterdam (UvA and AMC) on February 25, 2026 from 145.117.198.035
Copyright © 2026 American Society of Clinical Oncology. All rights reserved.

APPENDIX 1. METHODS

Study Design

This study was a post hoc analysis of the REVEAL study, a multicenter prospective observational cohort study.[8] We reviewed the clinical records of patients with colorectal cancer who underwent surgical resection between July 2015 and December 2021 at one of the three participating centers in the Netherlands: Maastricht University Medical Center, Zuyderland Medical Center, and VieCuri Medical Center. Patients with stage I to III disease who had undergone macroscopic complete resection—defined as either R0 (histopathologic tumor–free margins) or R1 (microscopically positive margin)—were included. Exclusion criteria were benign lesions, neuroendocrine tumors, progressive locoregional disease (within 30 days), or synchronous distant metastasis (within 90 days). The study protocol was approved by the Medical Ethical Committee of the Maastricht University Medical Center (ClinicalTrials.gov identifier: NCT02347735). A written informed consent was obtained from all participating patients.

Data Collection

We obtained the following data from the medical records: age, sex, BMI, pathological TNM stage, tumor characteristics, and follow-up data. Tumor characteristics included differentiation, extramural venous invasion, and tumor size as described in Table 1. Follow-up data included the development of recurrence, recurrence date and location, and date of the last outpatient clinic visit.

Sampling

Fecal samples were collected at the last outpatient clinic visit before surgery or at the day of hospital admission and stored for processing within 24 hours of collection at −80°C at the corresponding study sites.

Follow-Up

Surveillance included physical examinations and blood tests (including serum tumor markers) every 3 months for 3 years, followed by every 6 months thereafter. Imaging, including abdominal computed tomography (CT) or ultrasonography and chest CT, was performed every 6 months for 3 years and then annually. Total colonoscopy was conducted at 1 and 3 years postsurgery.

End Points and Definitions

This study's primary end point was the overall recurrence rate. The secondary end points were locoregional and distant recurrence rates. Locoregional recurrence was defined as newly diagnosed tumor after 30 days postsurgery, at the site of the anastomosis, regional lymph nodes, abdominal wall, cicatrix, tumor bed, vaginal wall, or the pelvic bone in relation to resected colon or rectal segments. Distant recurrence was defined as newly diagnosed tumor after 90 days postsurgery in all other sites, including the peritoneum and nonregional lymph nodes. Patients with combined locoregional and distant recurrence at presentation were classified as locoregional recurrence. Histopathologic verification of radiologic findings of metastases during follow-up was not needed if the multidisciplinary team conference deemed the finding as a recurrence. Time to detection of recurrence was defined as the interval from the date of surgery to the first radiologic detection of recurrence. Patients were classified as recurrence-free only if a minimum follow-up period of 3 years was completed. After this period, patients were censored at the date of their last outpatient clinic visit.

DNA Isolation

DNA was extracted from 150 mg of fecal samples using mechanical lysis and the PSP Spin Stool DNA Plus Kit (Cat No. 1038110300, STRATEC Molecular GmbH, Germany). Each sample was placed in Lysing Matrix E tubes along with 1,400 μL of stool stabilizer from the PSP kit. Mechanical lysis was performed with the FastPrep system in three cycles of 30 seconds at 6.5 m/s, with 30-second cooling intervals on ice in between each cycle. After lysis, the samples were heated at 95°C for 15 min, cooled on ice for 1 minute, and then centrifuged at 13,400 *g* for 1 minute. The resulting supernatant was transferred to the PSP InviAdsorb (Invitek Diagnostics, Tondela, Portugal) for further processing. Negative extraction controls, consisting of DNA-free water, were processed similarly. DNA concentrations were measured using a Nanodrop 1000 spectrophotometer (Thermo Fisher Scientific Inc, Waltham, MA).

16S rRNA Gene Sequencing

Amplicons targeting the V3-V4 region of the 16S rRNA gene were generated using a single-step polymerase chain reaction (PCR) protocol with primers (F341/R80). The PCR was performed under the following thermocycling conditions: initial denaturation at 98°C, followed by 25 cycles of denaturation (10 seconds at 98°C), annealing (20 sec at 55°C), extension (90 sec at 72°C), and final extension at 72°C for 10 min. PCR products were purified using AMpure XP beads (Cat No. A63882, Beckman Coulter Inc, Brea, CA) and then pooled in equimolar amounts. Sequencing was performed using a MiSeq platform with V3 chemistry in 2 × 251 cycles. Forward and reverse reads were truncated to 240 and 210 bases, respectively, and merged using USEARCH. Reads failing the Illumina chastity filter, with an expected error rate above two, or shorter than 380 bases were filtered out. Amplified sequence variants (ASVs) were inferred for each sample individually, with a minimum abundance of four reads. Unfiltered reads were mapped against the collective ASV set to determine the abundances, and taxonomy was assigned using SILVA database V132.

Statistical Analysis

Clinical metadata were analyzed using IBM SPSS Statistics Version 28.0. Descriptive statistics were used to summarize the study population. Categorical variables were expressed as frequency (percentage) and compared using the chi-square or Fisher's exact test. Continuous variables were expressed as medians with interquartile ranges and analyzed using the Mann-Whitney U test.

All statistical analyses of the 16S rRNA gene sequencing–derived data were performed using R (v. 4.3.2, RStudio v. 2023.12.1 + 402), using the following packages: phyloseq (v. 1.46.0), vegan (v. 2.6.4), DESeq2 (v. 1.42.1), microbiomeMarker (v. 1.13.2), and stats (v. 4.3.2). Alpha diversity was examined at observed species richness and Shannon diversity index level and compared between the groups using the Wilcoxon signed-rank test. Microbial composition was assessed using principal coordinate analysis at the ASV level based on unweighted UniFrac distance, which considers phylogenetic distance between bacterial taxa through presence/absence. Nonparametric permutational multivariate analysis of variance was applied using the vegan Adonis function on the distance matrix. Differences in genus relative abundances between groups were found using the DESeq method and confirmed using the linear discriminant analysis effect size (LEfSe) method.

For the differential abundance analysis, all genera were included in the model without any prevalence or abundance filtering. Analysis was performed separately for the two key comparisons: (a) locoregional recurrence vs. no recurrence (21 *v* 233 patients) and (b) distant recurrence versus no recurrence (40 *v* 233 patients). Differential abundance was assessed using the Wald test within the DESeq2 framework. Statistical significance was defined using Benjamini-Hochberg-adjusted $P < .05$, and a threshold of |log2 fold change| > 2 was applied to define biologically meaningful results. The eight genera shown in Figure 1E are those that met both criteria in at least one of the two comparisons. The results from the differential analysis were cross-referenced using the LEfSe approach on CPM-normalized data, applying a Kruskal-Wallis test *P*-value cutoff of .05 and reporting taxa with an linear discriminant analysis score of > 2.0.

Thresholds Definition

The relative abundance of *Porphyromonas* and *Anaeroplasma* was calculated as a percentage to the total reads of the matrix count table at the genus level. The top 10% of all samples was categorized as being in the high group, whereas the rest 90% was pooled together in the low group. In detail, the top 10% of all samples had a relative abundance of *Porphyromonas* and *Anaeroplasma* higher than 0.03% and 0.10%, respectively (Appendix Figs A1C and A1D). This stringent cutoff was chosen to identify patients with distinctly elevated abundance while pooling the remainder into a single group, preserving statistical power in this relatively small cohort. In previous work, we used the top/bottom 25% quantiles (Zafeiropoulou K, et al: Sci Rep 15: 26237, 2025); however, given the smaller sample size here, a narrower cutoff was applied to maximize interpretability and maintain clinical feasibility. We acknowledge that these thresholds are based on relative abundance and may vary across cohorts and sequencing platforms, underscoring the need for future studies to establish standardized, absolute abundance measures using quantitative approaches such as microbial cell counts or spike-in controls. Recurrence risk was compared between the high and low groups using Kaplan-Meier and Cox regression analyses and reported as HR and corresponding 95% CIs.

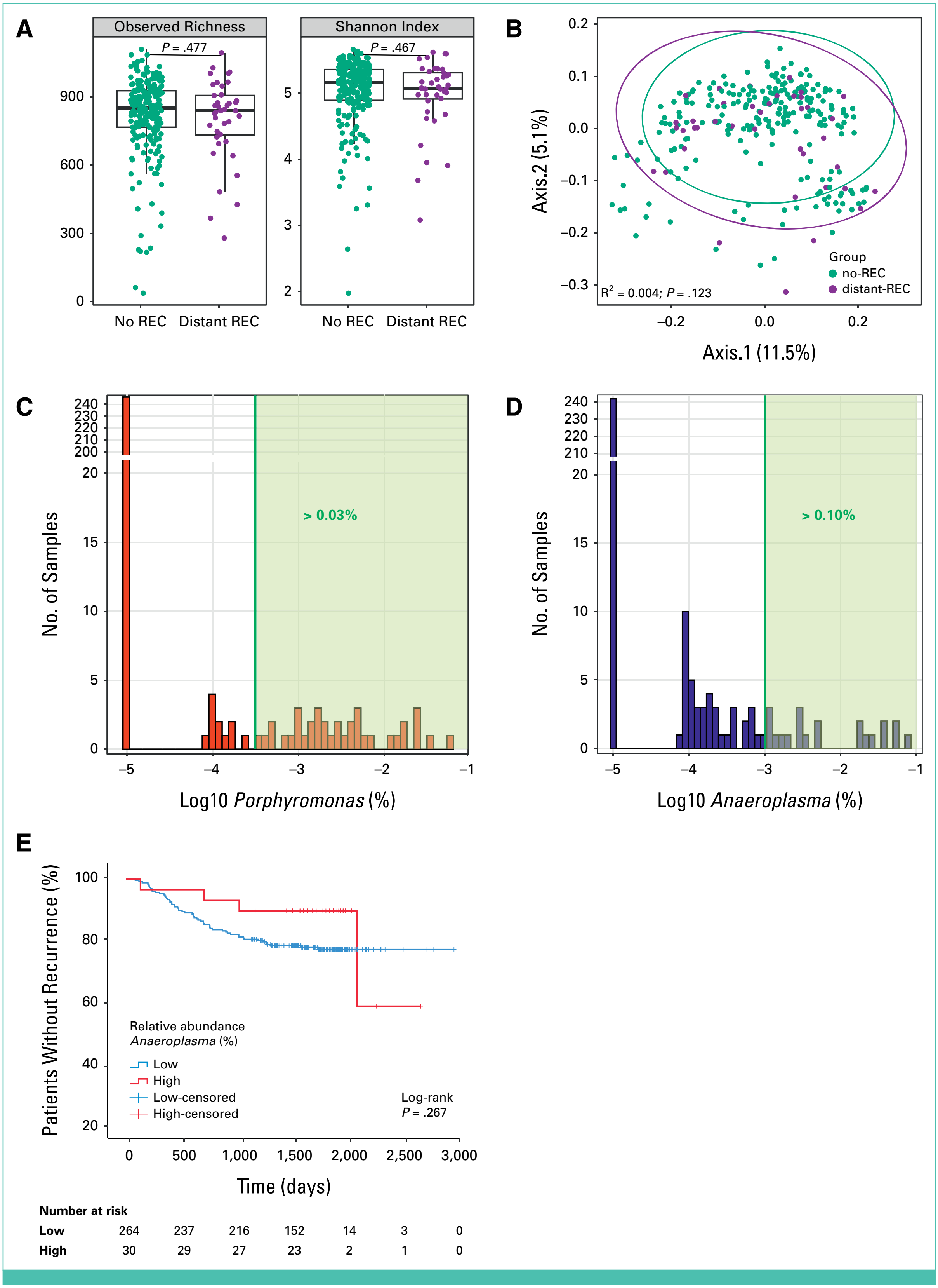

FIG A1. (A) No difference in alpha diversity between patients with only distant recurrence and patients without recurrence. Dots represent preoperative fecal samples, colored by recurrence status. (B) Patients with only distant recurrence do not cluster different from those without recurrence. (C and D) Density distribution plots of the relative abundance of *Porphyromonas* and *Anaeroplasma*, respectively. A small pseudocount (1e-5) was added before log transformation to include zero-abundance samples in the distribution. (E) Kaplan-Meier curve of overall recurrence risk based on high or (continued on following page)

Downloaded from ascopubs.org by Universiteit van Amsterdam (UvA and AMC) on February 25, 2026 from 145.117.198.035
Copyright © 2026 American Society of Clinical Oncology. All rights reserved.

© 2026 by American Society of Clinical Oncology

FIG A1. (Continued). low preoperative fecal abundance of *Anaeroplasma*. Vertical marks on the curve indicate censored patients at the end of the follow-up period. All REC, patients with recurrence; local REC, patients with locoregional recurrence; no REC, patients without recurrence; REC, recurrence. *P-adjusted < .05; **P-adjusted < .01; ***P-adjusted < .001.

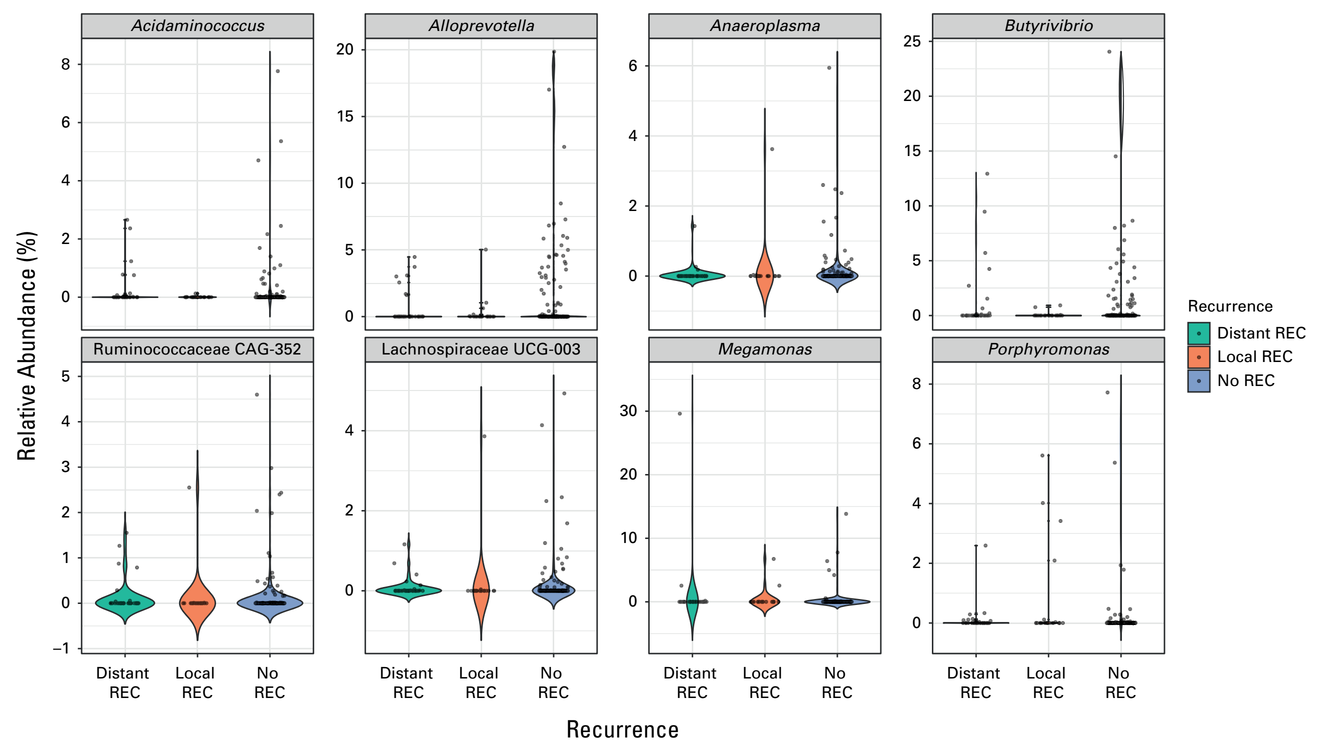

FIG A2. Violin plots showing the relative abundance distributions of the eight genera identified as differentially abundant and illustrated in Figure 1E. The distributions are shown separately for the three groups: distant recurrence (green), locoregional recurrence (orange), and no recurrence (blue). distant REC, patients with distant recurrence; no REC, patients without recurrence; local REC, patients with locoregional recurrence; REC, recurrence.

Downloaded from ascopubs.org by Universiteit van Amsterdam (UvA and AMC) on February 25, 2026 from 145.117.198.035
Copyright © 2026 American Society of Clinical Oncology. All rights reserved.

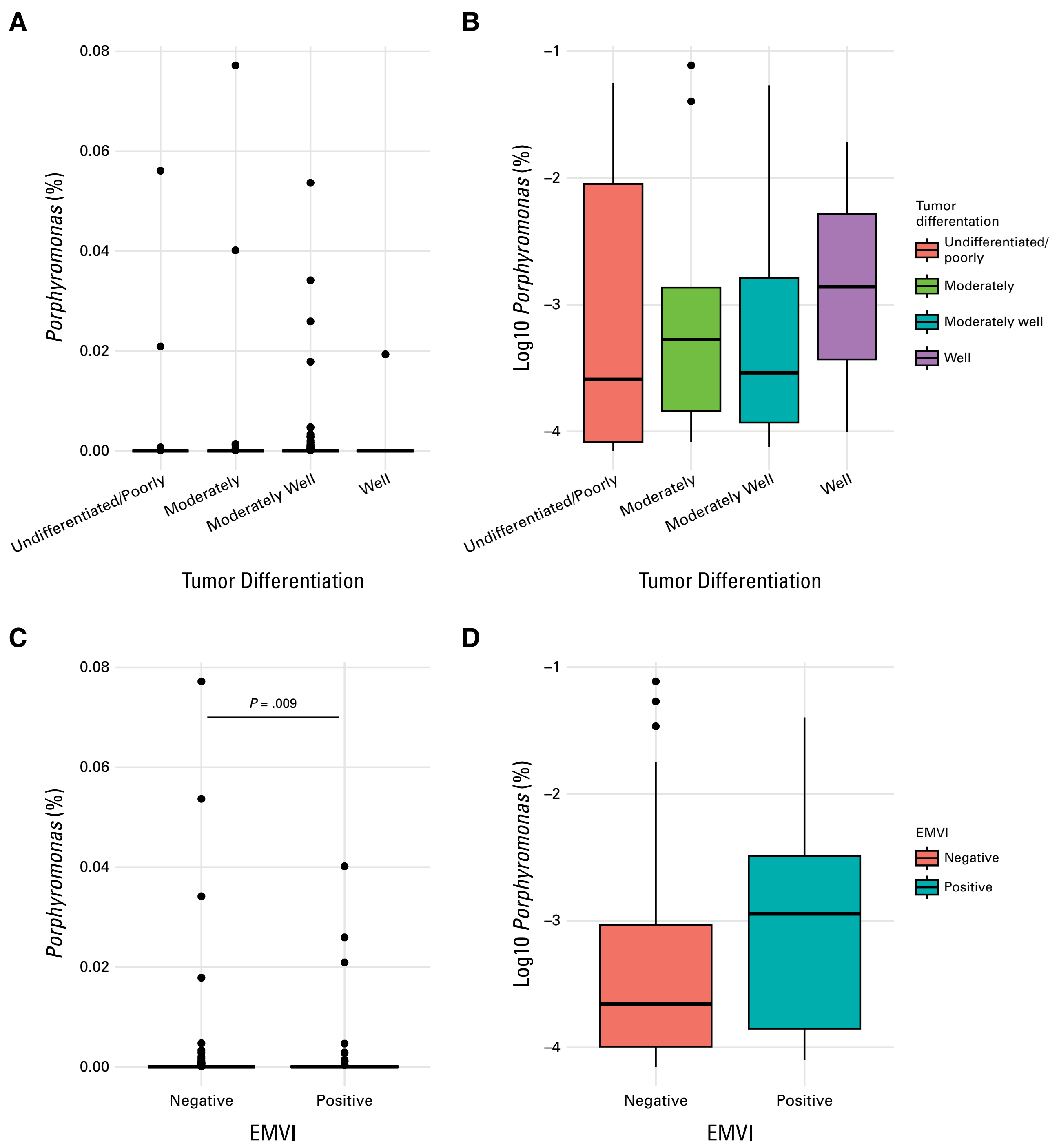

FIG A3. (A) Scatter and (B) box plots of relative *Porphyromonas* abundance across tumor differentiation grades. (C) Scatter and (D) box plots of *Porphyromonas* abundance in EMVI-negative versus EMVI-positive tumors. EMVI, extramural venous invasion.

Downloaded from ascopubs.org by Universiteit van Amsterdam (UvA and AMC) on February 25, 2026 from 145.117.198.035
Copyright © 2026 American Society of Clinical Oncology. All rights reserved.
